# Declining trends in exposures to harmful policing among people who inject drugs in Vancouver, Canada

**DOI:** 10.7448/IAS.19.4.20729

**Published:** 2016-07-18

**Authors:** Adina Landsberg, Thomas Kerr, Michael-John Milloy, Huiru Dong, Paul Nguyen, Evan Wood, Kanna Hayashi

**Affiliations:** 1 British Columbia Centre for Excellence in HIV/AIDS, St. Paul’s Hospital, Vancouver, BC, Canada; 2 Faculty of Health Sciences, McMaster University, Hamilton, ON, Canada; 3 Department of Medicine, St. Paul’s Hospital, University of British Columbia, Vancouver, BC, Canada

**Keywords:** harm reduction, HIV/AIDS, injection drug use, drug law enforcement, Canada, epidemiology

## Abstract

**Introduction:**

In 2006, the Vancouver Police Department (VPD) developed an organization-wide drug policy approach, which included endorsing harm reduction strategies for people who inject drugs (PWID). We sought to examine rates of potentially harmful policing exposures and associated HIV risk behaviour among PWID in Vancouver, Canada before and after the VPD policy change.

**Methods:**

Data were derived from two prospective cohort studies of PWID. Multivariable generalized estimating equation models were used to examine changes in the risk of confiscation of drug use paraphernalia and physical violence by the police, as well as changes in the relationship between exposures to the two policing practices and sharing of drug use paraphernalia, before and after the policy change.

**Results:**

Among 2193 participants, including 757 (34.5%) women, the rates of experiencing police confiscation of drug use paraphernalia declined from 22.3% in 2002 to 2.8% in 2014, and the rates of reporting experiencing physical violence by the police also declined from 14.1% in 2004 to 2.9% in 2014. In multivariable analyses, the post-policy change period remained independently and negatively associated with reports of confiscation of drug use paraphernalia (adjusted odds ratio (AOR): 0.25; 95% confidence interval (CI): 0.21 to 0.31) and reported physical violence by the police (AOR: 0.76; 95% CI: 0.63 to 0.91). However, experiencing both confiscation of drug use paraphernalia and physical violence by the police (AOR: 1.92; 95% CI: 1.10 to 3.33) and experiencing only confiscation of drug use paraphernalia (AOR: 1.71; 95% CI: 1.34 to 2.19) remained independently and positively associated with sharing of drug use paraphernalia during the post-policy change period.

**Conclusions:**

In our study, two policing practices known to increase HIV risk among PWID have declined significantly since the local police launched an evidence-based drug policy approach. However, these practices remained independently associated with elevated HIV risk after the post-policy change. Although there remains a continued need to ensure that policing activities do not undermine public health efforts, these findings demonstrate that a major shift towards a public health approach to policing is possible for a municipal police force.

## Introduction

In many settings, intensive policing is used as a common strategy aimed at eradicating the trafficking and use of illicit drugs [1,2]. However, a large body of evidence demonstrates that exposure to various policing practices increases HIV risk behaviours and other harms among people who inject drugs (PWID) [1–7]. Intensive policing practices, such as drug crackdowns, have been shown to elicit fear among PWID, promote risk behaviours [1–3,5,7–9] (such as sharing of used syringes [1,2,10]) and limit access to healthcare and essential HIV prevention services [1,7,11–13]. The downstream health consequences of these behaviours include increased risk of infection, both bacterial and viral, vascular damage and disease transmission [1,7]. Other specific policing practices, including confiscation of syringes [6,11] and arrest for syringe possession [14], have also been identified as perpetuating HIV risk among PWID. As well, previous studies have reported high rates of police-perpetrated violence among PWID [4,9,15], and such experiences have also been shown to increase fear of police and high-risk injection behaviour [4,15].

In response to growing concerns regarding the negative impacts of high-intensity policing targeting PWID, in recent years, police departments in some jurisdictions have sought to develop more progressive drug policies, including those with a focus on or acceptance of harm reduction approaches. Although evaluation of such novel policing policies and programmes is of great importance, there is a limited body of research on police-endorsed harm reduction strategies and their effect on the behaviours and health of PWID. For example, a study conducted in Tehran, Iran, sought to examine PWID's access to harm reduction programmes after the local government implemented harm reduction strategies in 2002 [16]; however, it did not examine their HIV serostatus or their exposure to policing. In Kyrgyzstan in 2009, a new policy was adopted to advise the police to not interfere with syringe exchange programmes and outreach to PWID and sex workers [17]. Although the study examined police awareness of the policy and related activities, it did not evaluate the effect the policy had on PWID.

In Vancouver, Canada, a large-scale police crackdown in 2003 targeting people who use drugs led to increase high-risk injection behaviours and displacement of local PWID and invited widespread criticism from public health and human rights experts [18,19]. Soon after, the Vancouver Police Department (VPD) launched a new drug policy approach in 2006, which consists of four pillars: prevention, enforcement, harm reduction and treatment [20]. With regard to harm reduction, the VPD stated that their public safety mission aims to “ensure open and ready access to public health harm reduction initiatives, such as needle exchange and the Supervised Injection Site” [20]. Although the policy document did not specify which policing practices should be avoided or encouraged, one would expect a reduction of harmful policing practices that are known to increase the risk of blood-borne disease transmission among PWID, such as confiscation of drug use paraphernalia and physical violence by the police [4,6,11,15]. Therefore, taking advantage of two long-running prospective cohort studies of PWID, we sought to examine changes in the risk of exposure to confiscation of drug use paraphernalia and physical violence by the police and the associated HIV risk behaviours among PWID in Vancouver, Canada, before and after the policy change in 2006.

## Methods

### Study procedures and participants

We pooled participants in two open prospective cohorts of people who use drugs in Vancouver: the Vancouver Injection Drug Users Study (VIDUS) and the AIDS Care Cohort to Evaluate Exposure to Survival Services (ACCESS). The cohorts have been described in detail elsewhere [21,22]. Briefly, VIDUS is a cohort of HIV-seronegative adult PWID who injected illicit drugs in the month prior to enrolment. ACCESS is a cohort of HIV-seropositive adult drug users who used an illicit drug other than cannabis in the previous month at enrolment. Other common eligibility criteria included being aged 18 years or older, residing in the greater Vancouver area and providing written informed consent. The two studies employ harmonized data collection and follow-up procedures to allow for combined analyses. Specifically, at baseline and semi-annually thereafter, participants answer an interviewer-administered questionnaire, which elicits data on demographic characteristics, drug-using behaviours and related exposures, and undergo HIV serologic testing or disease monitoring as appropriate. Participants received $30 CAD at study visits. Both studies have been approved by the University of British Columbia/Providence Healthcare Research Ethics Board.

For the present analyses, participants were eligible if they completed at least one study visit between 1 June 2002 and 30 November 2014, reported a history of injection drug use at baseline, and reported having injected drugs or smoked crack cocaine during the previous six months for each interview.

### Study variables

For the examination of the trends in the risk of policing exposures, there were two primary outcomes: experiencing confiscation of drug use paraphernalia (i.e. new syringes and pipes) by the police in the previous six months (yes *vs*. no) and experiencing physical violence by the police in the previous six months (yes *vs*. no). For the examination of the associated HIV risk, the primary outcome was sharing drug use paraphernalia (i.e. syringes and pipes) in the previous six months (yes *vs*. no). In addition to syringes, we included pipes in the variable definition, as previous studies have shown increasing trends in crack smoking and the associated elevated risk of HIV seroconversion among PWID in this setting [23,24].

For the examination of the trends in the risk of policing exposures, the primary explanatory variable was the estimated calendar year of the outcome, dichotomized into before and after the VPD policy change in 2006. The study questionnaire assessed the outcomes of interest occurring in the past six months, so the calendar year was estimated as the year of the date occurring three months prior to the interview date. Because the reports of police confiscation of drug use paraphernalia were assessed only between June 2002 and May 2006 and again between June 2009 and November 2014 (i.e. the question was removed for administrative purposes between June 2006 and May 2009), the variable was dichotomized as 2009 to 2014 *versus* 2002 to 2006 for the analysis of police confiscation of drug use paraphernalia. Similarly, the reports of physical violence by the police were assessed only between June 2004 and November 2014, and therefore the variable was dichotomized as 2007 to 2014 *versus* 2004 to 2006 for the analysis of physical violence by the police. For the examination of the relationship between exposure to the two policing practices and sharing of drug use paraphernalia, the primary explanatory variable was exposures to the two policing practices in the previous six months. This variable had four categories: (1) experiencing both confiscation of drug use paraphernalia and physical violence by the police; (2) experiencing only confiscation of drug use paraphernalia by the police; (3) experiencing only physical violence by the police; and (4) experiencing neither of them.

Based on existing literature [4,6,15,25], we considered secondary explanatory variables that might confound the relationships between the primary explanatory variables and the outcomes. These included: age (in years); gender (male *vs*. female); ancestry (Caucasian *vs*. other); homelessness (yes *vs*. no); Downtown Eastside residence (yes *vs*. no); heroin injection (≥daily *vs*. <daily); cocaine injection (≥daily *vs*. <daily); crack smoking (≥daily *vs*. <daily); injection of drugs in public (yes *vs*. no); drug dealing (yes *vs*. no); sex work involvement (yes *vs*. no); incarceration (yes *vs*. no); and HIV serostatus (positive *vs*. negative). Behavioural variables referred to the previous six months unless otherwise indicated and were treated as time-varying variables.

### Statistical analyses

First, we examined the baseline sample characteristics stratified by reports of policing exposures in the previous six months, using the Pearson's chi-square test (for categorical variables) and Wilcoxon rank-sum test (for continuous variables). We also plotted the proportions of participants reporting confiscation of drug use paraphernalia and physical violence by the police in the previous six months over the calendar year. Because our questionnaire asked about police confiscation of drug use paraphernalia during the past month between 2006 and 2014, we added the past month data to the plot.

Because the present analyses included serial measures for each participant, we used generalized estimating equations (GEE) with logit link, which provided standard errors adjusted by multiple observations per person using an exchangeable correlation structure. As a first step, we fitted univariable GEE models to examine the unadjusted associations between the explanatory variables and the outcomes. To determine whether the calendar year after the VPD policy change was associated with decreased risk of exposures to the two policing practices after adjustment for potential confounders, we used an *a priori*-defined statistical protocol [26] to construct multivariable GEE models. Briefly, we first built the full multivariable GEE models for each of the two outcomes, which included all explanatory variables associated with the outcome at *p*<0.05 in the univariable models. Then, we fit a series of reduced models comparing the coefficient value associated with the primary explanatory variable in the full model to its corresponding value in each of the reduced models and dropped the secondary explanatory variables associated with the smallest relative change. We continued this iterative process until the minimum change exceeded 5%.

Next, to identify changes in the relationship between exposures to the two policing practices and sharing of drug use paraphernalia before and after the VPD policy change, we first used data from throughout the study period to build a multivariable GEE model, employing the same statistical protocol described above. Then, we divided the study period into two sub-periods (June 2004 to May 2006 and June 2009 to November 2014) based on the timing of the VPD policy change, as well as the availability of the required data, and fit a multivariable model for each of the two periods. The two models included the same set of primary and secondary explanatory variables, allowing us to compare the effect estimates for the primary explanatory variable between the two periods.

We also used descriptive statistics to examine the following: the proportion of participants who reported that drug use paraphernalia were returned to them after having been confiscated by the police; types of physical violence by the police that participants reported experiencing; and what participants reported doing immediately before experiencing physical violence by the police. The analysis was restricted to a period of June 2009 and November 2014 because these sub-questions were added to the questionnaire in June 2009. All *p*-values were two-sided. All statistical analyses were performed using the SAS software version 9.4 (SAS Institute, Cary, NC).

## Results

### Sample characteristics

In total, 2193 participants were eligible for the present analyses, including 757 (34.5%) women. Of these, median age at baseline was 40 years (interquartile range (IQR): 32 to 46), and 60.0% self-reported having Caucasian ancestry. A total of 19,027 interviews were conducted, with a median of 7 (IQR: 3 to 13) interviews per person. A total of 179 participants were not asked about police confiscation of drug use paraphernalia, whereas 109 participants were not asked about police physical violence. As shown in Table 1, 242 (12.0%) of 2014 participants reported experiencing police confiscation of drug use paraphernalia, 186 (8.9%) of 2084 participants reported experiencing physical violence by the police, and 1279 (58.3%) of 2193 participants reported having shared drug use paraphernalia during the previous six months at their respective baseline periods. For the analyses of police confiscation of drug use paraphernalia, 1698 (84.3%) of 2014 participants were followed during both periods (2002 to 2006 and 2009 to 2014), and the baseline rate of reporting police confiscation (12.2%) was not statistically different from that (11.1%) among those followed in either period only (*p*=0.576). Similarly, for the analyses of police violence, 1780 (85.4%) of 2084 participants were followed during both periods (2004 to 2006 and 2007 to 2014), and the baseline rate of reporting police violence (8.9%) was essentially the same as that (8.9%) among those followed in either period only (*p*=0.977).

**Table 1:** Baseline sample characteristics stratified by reports of confiscation of drug use paraphernalia and physical violence by the police in the previous six months among PWID in Vancouver, Canada (*n*=2193)

		Police confiscation of drug use paraphernalia^a^,^b^			Police physical violence^a^,^c^		
					
Characteristic	Total, *n* (%)	Yes *n* (%) 242 (12.0)	No *n* (%) 1772 (88.0)	*p*	Yes *n* (%) 186 (8.9)	No *n* (%) 1898 (91.1)	*p*
Demographic							
Age (median, IQR)	40 (32 to 46)	36 (28 to 43)	42 (34 to 47)	<0.001	37 (31 to 43)	41 (34 to 47)	<0.001
Male gender	1436 (65.5)	154 (63.6)	1158 (65.4)	0.600	134 (72.0)	1234 (65.0)	0.054
Caucasian ancestry	1316 (60.0)	129 (53.3)	1076 (60.7)	0.027	123 (66.1)	1126 (59.3)	0.071
Homeless^a^	682 (31.1)	93 (38.4)	436 (24.6)	<0.001	102 (54.8)	554 (29.2)	<0.001
DTES residence^a^	1391 (63.4)	163 (67.4)	1065 (60.1)	0.030	124 (66.7)	1229 (64.8)	0.602
≥Daily injection heroin use^a^	640 (29.2)	121 (50.0)	402 (22.7)	<0.001	78 (41.9)	524 (27.6)	<0.001
≥Daily injection cocaine use^a^	337 (15.4)	64 (26.5)	241 (13.6)	<0.001	39 (21.0)	250 (13.2)	0.003
≥Daily crack smoking^a^	892 (40.7)	159 (65.7)	605 (34.1)	<0.001	98 (52.7)	742 (39.1)	<0.001
Injected drugs in public^a^	882 (40.2)	153 (63.2)	559 (31.5)	<0.001	104 (55.9)	655 (34.5)	<0.001
Drug dealing^a^	649 (29.6)	119 (49.2)	395 (22.3)	<0.001	97 (52.2)	498 (26.2)	<0.001
Sex work^a^	371 (16.9)	55 (22.7)	263 (14.8)	0.002	27 (14.5)	295 (15.5)	0.700
Incarceration^a^	370 (16.9)	85 (35.1)	201 (11.3)	<0.001	71 (38.2)	260 (13.7)	<0.001
HIV positive^a^	831 (37.9)	62 (25.6)	720 (40.6)	<0.001	68 (36.6)	731 (38.5)	0.593
Sharing of drug use paraphernalia^a^	1279 (58.3)	181 (74.8)	864 (48.8)	<0.001	125 (67.2)	1031 (54.3)	0.001

PWID: people who inject drugs; IQR: interquartile range; DTES: Downtown Eastside; HIV: human immunodeficiency virus

a denotes activities in the previous six months

b for this analysis, *n*=2014

c for this analysis, *n*=2084.

### Trends in police confiscation of drug use paraphernalia and physical violence

In total, 528 (26.2%) of 2014 participants reported experiencing police confiscation of drug use paraphernalia at least once, and 472 (22.6%) of 2084 participants reported experiencing physical violence by the police at least once during their respective study periods. After June 2009, there were 277 reports of police confiscation of drug use paraphernalia, and the paraphernalia were reportedly returned to participants only on three (1.1%) occasions. There were 283 reports of physical violence by the police after June 2009. Of these, the most commonly reported types of physical violence experienced included the following: bruises (41.0%), scratches (20.5%) and broken bones (6.7%). Prior to experiencing physical violence by the police, participants most commonly reported engaging in the following activities: nothing (30.7%), selling drugs (8.1%) and criminal activity (7.1%).

**Figure 1:**
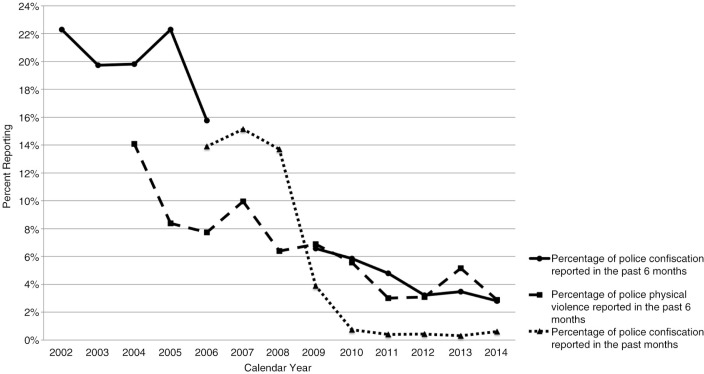
Rates of reporting confiscation of drug user paraphernalia and physical violence by the police among PWID in Vancouver, Canada (*n*=2193).

As shown in Figure 1, the rates of experiencing police confiscation of drug use paraphernalia declined from 22.3% in 2002 to 2.8% in 2014, and the rates of experiencing physical violence by the police also declined from 14.1% in 2004 to 2.9% in 2014.

Table 2 presents the results of univariable and multivariable GEE analyses of changes in the risk of experiencing the two policing practices before and after the VPD policy change. As shown, in the final multivariable models, the post-policy change period remained independently and negatively associated with reports of confiscation of drug use paraphernalia (adjusted odds ratio (AOR): 0.25; 95% confidence interval (CI): 0.21 to 0.31) and physical violence by the police (AOR: 0.76; 95% CI: 0.63 to 0.91).

**Table 2:** Univariable and multivariable GEE analyses of factors associated with confiscation of drug use paraphernalia and physical violence by the police before and after the VPD policy change among PWID in Vancouver, Canada (*n*=2193)

	Police confiscation of drug use paraphernalia^a^,^b^		Physical violence by the police^a^,^c^	
		
Characteristic	Unadjusted OR (95% CI)	Adjusted OR (95% CI)	Unadjusted OR (95% CI)	Adjusted OR (95% CI)
Calendar year (2009 to 2014 *vs*. 2002 to 2006) (2007 to 2014 *vs*. 2004 to 2006)	0.18 (0.15 to 0.21)	0.25 (0.21 to 0.31)	0.56 (0.48 to 0.65)	0.76 (0.63 to 0.91)
Gender (Male *vs*. female)	0.93 (0.76 to 1.14)		2.01 (1.59 to 2.54)	2.15 (1.68 to 2.75)
Age (Per 10-year increase)	0.43 (0.39 to 0.47)	0.72 (0.65 to 0.80)	0.56 (0.51 to 0.62)	0.68 (0.60 to 0.76)
Ancestry (Caucasian *vs*. other)	0.81 (0.67 to 0.99)		1.26 (1.02 to 1.56)	
Homelessness^a^ (Yes *vs*. no)	2.46 (2.08 to 2.89)	2.22 (1.88 to 2.63)	2.43 (2.05 to 2.88)	1.64 (1.37 to 1.96)
DTES residence^a^ (Yes *vs*. no)	1.13 (0.95 to 1.34)		1.37 (1.14 to 1.64)	
Heroin injection^a^ (≥Daily *vs*. <daily)	3.02 (2.56 to 3.56)		1.89 (1.58 to 2.25)	
Cocaine injection^a^ (≥Daily *vs*. <daily)	2.16 (1.79 to 2.61)		1.70 (1.37 to 2.12)	1.42 (1.13 to 1.79)
Crack smoking^a^ (≥Daily *vs*. <daily)	4.08 (3.48 to 4.80)	2.78 (2.36 to 3.27)	1.53 (1.31 to 1.80)	
Injected drugs in public^a^ (Yes *vs*. no)	3.82 (3.27 to 4.46)		2.49 (2.11 to 2.94)	
Drug dealing^a^ (Yes *vs*. no)	2.67 (2.29 to 3.12)		2.71 (2.33 to 3.16)	
Sex work^a^ (Yes *vs*. no)	2.20 (1.79 to 2.71)		1.16 (0.91 to 1.48)	
Incarceration^a^ (Yes *vs*. no)	4.81 (4.05 to 5.71)		4.74 (3.94 to 5.71)	3.31 (2.70 to 4.05)
HIV serostatus^a^ (Positive *vs*. negative)	0.61 (0.49 to 0.77)		0.67 (0.54 to 0.82)	0.82 (0.67 to 1.02)

GEE: generalized estimating equations; VPD: Vancouver Police Department; PWID: people who inject drugs; OR: odds ratio; CI: confidence interval; DTES: Downtown Eastside.

a Denotes activities in the previous six months

b for this analysis, *n*=2014

c for this analysis, *n*=2084.

### Changes in the association with HIV risk behaviour

Table 3 shows the results of multivariable GEE analyses of the relationship between exposures to policing and sharing of drug use paraphernalia, stratified by two sub-periods. As shown, in 2004 to 2006, experiencing confiscation of drug use paraphernalia but not physical violence by the police remained independently and positively associated with sharing of drug use paraphernalia (AOR: 1.37; 95% CI: 1.02 to 1.85). In 2009 to 2014, experiencing both confiscation of drug use paraphernalia and physical violence by the police (AOR: 1.92; 95% CI: 1.10 to 3.33) and experiencing confiscation of drug use paraphernalia but not physical violence by the police (AOR: 1.71; 95% CI: 1.34 to 2.19) remained independently and positively associated with sharing of drug use paraphernalia.

**Table 3:** Multivariable GEE analyses of the relationship between exposures to policing and sharing of drug use paraphernalia among PWID in Vancouver, Canada (*n*=2193)

	2004 to 2006^b^	2009 to 2014^c^
	
Characteristic	AOR (95% CI)	AOR (95% CI)
Exposures to policing^a^ (Both confiscation of drug use paraphernalia and physical violence by the police *vs*. neither) (Confiscation of drug use paraphernalia but not physical violence by the police *vs*. neither) (Physical violence but not confiscation of drug use paraphernalia by the police *vs*. neither)	0.96 (0.57 to 1.60) 1.37 (1.02 to 1.85) 1.03 (0.66 to 1.62)	1.92 (1.10 to 3.33) 1.71 (1.34 to 2.19) 1.13 (0.85 to 1.50)
Age (Per 10-year increase)	1.10 (0.99 to 1.22)	0.81 (0.74 to 0.88)
Homelessness^a^ (Yes *vs*. no)	1.29 (1.00 to 1.67)	1.34 (1.18 to 1.52)
Heroin injection^a^ (≥Daily *vs*. <daily)	0.74 (0.60 to 0.92)	0.94 (0.81 to 1.08)
Crack smoking^a^ (≥Daily *vs*. <daily)	2.14 (1.76 to 2.60)	1.98 (1.76 to 2.22)
Injected drugs in public^a^ (Yes *vs*. no)	1.70 (1.33 to 2.16)	1.70 (1.51 to 1.91)
Drug dealing^a^ (Yes *vs*. no)	1.47 (1.18 to 1.83)	1.33 (1.17 to 1.50)
Sex work^a^ (Yes *vs*. no)	1.35 (1.04 to 1.75)	1.43 (1.20 to 1.70)
Incarceration^a^ (Yes *vs*. no)	1.75 (1.32 to 2.32)	1.10 (0.93 to 1.31)

GEE: generalized estimating equations; PWID: people who inject drugs; AOR: adjusted odds ratio; CI: confidence interval.

a Denotes activities in the previous six months

b for this analysis, *n*=1012

c for this analysis, *n*=1494.

## Discussion

We found that approximately one-quarter of participants experienced confiscation of drug use paraphernalia or physical violence by the police, respectively, at least once during the 12-year study period. Post-VPD policy change, there was a significant decline in the prevalence of experiencing police confiscation of drug use paraphernalia, as well as physical violence by the police, after extensive confounder adjustment. However, experiencing both confiscation of drug use paraphernalia and physical violence by the police, and experiencing confiscation of drug use paraphernalia but not physical violence by the police, remained independently and positively associated with sharing of drug use paraphernalia during the post-policy change period. Additionally, the effect size of the association between exposure to harmful policing and sharing of drug use paraphernalia appears to have increased after the VPD policy change.

Although the overall declining trends in exposure to harmful policing observed among our sample of PWID are encouraging, the persistent and seemingly stronger association between exposure to harmful policing and HIV risk behaviour during the post-VPD policy change period is concerning. In Vancouver, there has been a general decline in the rates of sharing of syringes and crack pipes during the last decade [27,28]. This decrease in HIV risk behaviour has coincided with greater and easier access to sterile drug use paraphernalia, as a result of decentralization of needle exchange programmes (NEPs) that led to widespread syringe distribution [27,29], and the launch and scale-up of crack pipe distribution programmes beginning in 2004 [30]. Now that there is greater coverage of NEPs and crack pipe distribution programmes in this setting, it may be that competing risks of sharing drug use paraphernalia (e.g. requiring many sterile syringes due to high-intensity drug use) have decreased in recent years and, consequently, exposure to harmful police activities may have had a greater effect on this behaviour among PWID.

Our findings suggest that between the two policing practices examined in this study, exposure to confiscation of drug use paraphernalia by the police appears to be the major factor associated with elevated HIV risk behaviour throughout the study period. The result that experiencing both types of policing practices was not independently associated with HIV risk behaviour during the 2004 to 2006 period may be due to the statistical power or may suggest that that may be the case. Of the individuals who experienced confiscation of drug use paraphernalia after June 2009 in our study, only about 1% reported having their paraphernalia returned to them by the police. Thus, sharing of drug use paraphernalia may be a direct consequence of confiscation. On the contrary, physical violence by the police has been shown to provoke fear in PWID [4,15] and increase apprehension of being stopped by the police [10], thus making PWID more reluctant to carry sterile drug paraphernalia and therefore indirectly impacting their HIV risk behaviours.

We also found that one-third of the participants who reported experiencing physical violence by the police after 2009 reported engaging in nothing prior to experiencing the violence. This finding is concerning, as it has been suggested that many PWID were exposed to unjustified, discriminatory abuse by the police during the police crackdown of 2003 in Vancouver [19]. Such human rights concerns have also been raised in many countries, including Thailand, Kazakhstan and China [15,31,32]. In Thailand, police have used visible track marks on the arms of PWID as an ostensible excuse to physically abuse or arrest them [33]. In our study, however, further in-depth investigation is needed to determine the context of police violence before any major inferences are made.

In addition to the VPD policy change in 2006, there has been a gradual scale-up of harm reduction services in this setting during the study period, which may have further promoted changes in policing practices [27,34]. Although we cannot make a causal conjecture from this observational study, we found that both of the harmful policing activities of interest have markedly decreased since 2006, suggesting that the VPD policy change may have served to positively change policing practices in this setting. These findings demonstrate that a significant shift of police attitudes towards harm reduction policies is possible. However, it remains important to explore potential reasons why these harmful behaviours still persist. Previous studies have demonstrated that police in some settings are misinformed of the law [17,35,36], whereas others are aware of the specific laws but continue to oppose them because progressive harm reduction policies may not align with their personal beliefs [35]. As we can only speculate about the reasons for the scarce yet persistent occurrence of these policing actions, in order to refine harm reduction training and implementation, the police should be further consulted [36]. In addition, the present harm reduction programmes in Vancouver must continue to be sustained, as police partnership with public health services, such as supervised injection facilities, has been shown to benefit PWID, increase public order and increase public support of these important facilities [37].

This study has several limitations. First, because the VIDUS and ACCESS are not random samples, the generalizability may be limited. Second, the self-reported data may be affected by response bias and socially desirable responding. However, previous research has shown that reported behaviours by PWID are generally truthful and reliable [38,39]. Third, the observational research study design may have excluded unmeasured confounding variables from consideration, although we did extensively adjust for potential confounding variables. Fourth, our questionnaire did not differentiate between the confiscation of syringes and pipes, and therefore the analyses could not be stratified to consider syringes and pipes separately as well as in combination. Last, future research should focus on the internal process within the police department and examine how the VPD policy change has been translated into street-level policing practices.

## Conclusions

We found a significant decrease in the proportion of PWID exposed to confiscation of drug use paraphernalia and physical violence by the police during the time period after the VPD drug policy change, compared to the time period before the drug policy change. Although it is encouraging that there is a significantly lower prevalence of exposure to these harmful policing methods, it is noteworthy that those who were exposed to these policing practices after the policy change were even more likely to engage in HIV risk behaviours. These findings suggest that overall the VPD may have been successful at adhering to the spirit of their drug policy; however, more could be done to protect PWID from harmful policing and associated HIV risk behaviours. Therefore, there is a need for further police engagement with harm reduction services to ensure that public health efforts have the greatest favourable impact on PWID and the public at large.
